# Using an electronic discharge notification system reduces the time delay between discharge and a summary being sent to the GP

**DOI:** 10.1192/bjo.2021.467

**Published:** 2021-06-18

**Authors:** Alex Adams, Bodvar Ymisson, Virginia Davies

**Affiliations:** South London and Maudsley NHS Foundation Trust

## Abstract

**Aims:**

All patients discharged from our Paediatric Liaison Team will have an electronic discharge summary sent to their GP within 24 hours by January 2020.

**Background:**

Writing a GP discharge summary is an essential part of patient care and is a patient safety issue if not completed on time. The NHS England Standard Contract states discharge summaries should be completed and sent to a GP within 24 hours of discharge. Baseline data showed our median time between discharge and a GP summary being sent off as 3 days and a baseline survey of staff in our team rated our discharge summary process as inefficient and time consuming. At baseline our discharge summary was typed on a word document which was then emailed to admin staff who would print and post to the GP. Our electronic patient record had an inbuilt discharge notification function that generates and sends summaries via email to the GP that other teams in the trust were already using.

**Method:**

We utilised the Model for Improvement Quality Improvement methodology. Initially we created a driver diagram breaking the process of discharge summary writing into its constituent components to generate change ideas. We then tested out these out in plan, do, study, act (PDSA) cycles whilst continually collecting data using a shared team spreadsheet to monitor for change.

**Result:**

We found that switching to electronically sent discharge notifications improved our time from discharge to a summary being sent to the GP from a median of 3 days to 1 day. We noticed that alongside a shared team spreadsheet monitoring when summaries were written we also reduced variation of time between discharge and a summary from a range of 0-27 days (with an outlier of 161) to 0-9 days.

**Conclusion:**

On average the time from discharge to a summary being written met the standard and we reduced the variability of time delay by using an electronic notification. However only 56% of summaries were sent within the 24 hour limit. Key factors for continued variability identified during regular team meetings included overall caseload of patients, amount of staff on shift and technical issues with the form. Our plan for sustainability is to discuss monthly in the team meeting any discharges that took longer than 1 day and target further PDSA cycles to these issues.

